# Neural Correlates of Auditory Figure-Ground Segregation Based on Temporal Coherence

**DOI:** 10.1093/cercor/bhw173

**Published:** 2016-08-30

**Authors:** Sundeep Teki, Nicolas Barascud, Samuel Picard, Christopher Payne, Timothy D. Griffiths, Maria Chait

**Affiliations:** 1 Wellcome Trust Centre for Neuroimaging, University College London, London WC1N 3BG, UK; 2 Auditory Cognition Group, Institute of Neuroscience, Newcastle University, Newcastle upon Tyne NE2 4HH, UK; 3 Ear Institute, University College London, London WC1X 8EE, UK; 4 Current address: Department of Physiology, Anatomy and Genetics, University of Oxford, Oxford OX1 3QX, UK

**Keywords:** auditory cortex, auditory scene analysis, intraparietal sulcus, magnetoencephalography, segregation, temporal coherence

## Abstract

To make sense of natural acoustic environments, listeners must parse complex mixtures of sounds that vary in frequency, space, and time. Emerging work suggests that, in addition to the well-studied spectral cues for segregation, sensitivity to temporal coherence—the coincidence of sound elements in and across time—is also critical for the perceptual organization of acoustic scenes. Here, we examine pre-attentive, stimulus-driven neural processes underlying auditory figure-ground segregation using stimuli that capture the challenges of listening in complex scenes where segregation cannot be achieved based on spectral cues alone. Signals (“stochastic figure-ground”: SFG) comprised a sequence of brief broadband chords containing random pure tone components that vary from 1 chord to another. Occasional tone repetitions across chords are perceived as “figures” popping out of a stochastic “ground.” Magnetoencephalography (MEG) measurement in naïve, distracted, human subjects revealed robust evoked responses, commencing from about 150 ms after figure onset that reflect the emergence of the “figure” from the randomly varying “ground.” Neural sources underlying this bottom-up driven figure-ground segregation were localized to planum temporale, and the intraparietal sulcus, demonstrating that this area, outside the “classic” auditory system, is also involved in the early stages of auditory scene analysis.”

## Introduction

A major challenge for understanding listening in the crowded environments we typically encounter involves uncovering the perceptual and neuro-computational mechanisms by which the auditory system extracts a sound source of interest from a hectic scene. Until recently, most such attempts focused primarily on “figure” and “ground” signals that differ in frequency, motivated by findings that segregation is associated with activation of spatially distinct populations of neurons in the primary auditory cortex (A1), driven by neuronal adaptation, forward masking, and frequency selectivity (for reviews, see: Fishman et al. 2001, 2004; Carlyon 2004; Micheyl, Carlyon, et al. 2007; Micheyl, Hanson, et al. 2007; Gutschalk et al. 2008; Elhilali, Ma, et al. 2009; Elhilali, Xiang, et al. 2009; Fishman and Steinschneider 2010; Kidd et al. 2011; Moore and Gockel 2012; Snyder et al. 2012).

**Figure 1.**
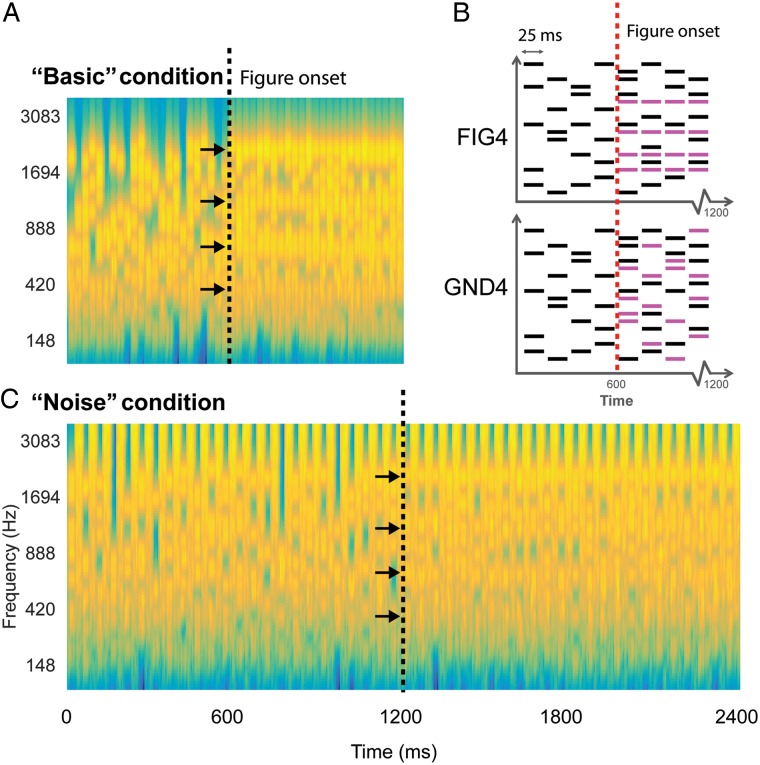
Stochastic figure-ground stimulus. (*A*) An example spectrogram of the basic SFG stimulus. Signals consisted of a sequence of 25 ms chords, each containing a random number of pure tone components that varied from 1 chord to the next. At 600 ms after onset (black dashed line), a certain number of components (coherence = 2, 4, or 8; 4 in this example; indicated by arrows) were fixed across chords in the second half of the stimulus. The resulting percept is that of a “figure” within a randomly varying background. (*B*) A schematic of the basic SFG stimulus whose spectrogram is shown in *A*. Randomly varying background chords (in black, 25 ms long) form the “no-figure” part of the stimulus. Following the transition (indicated by red dotted lines), 4 extra components (shown in pink) are added which are temporally correlated in the figure condition (FIG4), while randomly occurring in the ground condition (GND4). (*C*) The noise SFG stimulus is constructed similar to the basic SFG stimulus except for the introduction of 25 ms chords of white noise between each SFG chord. The plots in *A*,*C* represent “auditory” spectrograms, generated with a filterbank of 1/ERB wide channels (Equivalent Rectangular Bandwidth; Moore and Glasberg (1983)) equally spaced on a scale of ERB rate. Channels are smoothed to obtain a temporal resolution similar to the Equivalent Rectangular Duration (Plack and Moore 1990).

However, emerging work suggests that spectral separation per se is neither sufficient (Elhilali, Ma, et al. 2009) nor necessary (Teki et al. 2011, 2013; Micheyl, Kreft, et al. 2013; Micheyl, Hanson, et al. 2013; Christiansen et al. 2014; O'Sullivan et al. 2015) for segregation to take place. Using a broadband signal (“stochastic figure-ground”: SFG; Fig. 1), comprised of a sequence of brief chords containing random pure tone components that vary from 1 chord to another, we demonstrated that listeners are highly sensitive to the occasional repetition of a subset of tone-pips across chords. Perceptually, the repeating tones fuse together to form a “figure” that pops out from the randomly varying “ground” (Teki et al. 2011, 2013). This emergence of structure from a stochastic background captures the challenges of hearing in complex scenes where sources overlap in spectrotemporal dimensions such that segregation cannot be achieved based on spectral cues alone. The notable sensitivity exhibited by listeners confirms that the auditory system possesses specialized mechanisms which are tuned to the temporal coincidence of a small subset of sound elements within a mixture. The general pattern of performance, including that it scales with the number of temporally correlated channels, is consistent with the predictions of a recent model of auditory segregation—“temporal coherence model” (see extensive discussion in Shamma et al. 2011; Teki et al. 2013), based on a hypothesized mechanism that captures the extent to which activity in distinct neuronal populations that encode different perceptual features is correlated in time (Krishnan et al. 2014). The model proposes that, in addition to spectral separation, the auditory system relies on temporal relationships between sound elements to perceptually organize acoustic scenes (Elhilali, Ma, et al. 2009; Shamma et al. 2011; Micheyl, Hanson, et al. 2013; Micheyl, Kreft, et al. 2013).

Using fMRI, and an SFG signal that contained brief “figures” interspersed within long random tone patterns, we previously observed activations in planum temporale (PT), superior temporal sulcus (STS), and, intriguingly, in the intraparietal sulcus (IPS; Teki et al. 2011) evoked specifically by the appearance of temporally coherent tone patterns. However, due to the poor temporal resolution of fMRI, it remains unclear at what stage, in the course of figure-ground segregation, these areas play a role. In particular, a central issue pertains to whether activity in IPS reflects early processes that are causally responsible for segregation or rather the (later) consequences of perceptual organization (Cusack 2005; Shamma and Micheyl 2010; Teki et al. 2011).

The present magnetoencephalography (MEG) study was designed to capture the temporal dynamics of the brain regions involved in segregating the SFG stimulus. Participants performed an incidental visual task while passively listening (in separate blocks) to 2 versions of SFG signals (Fig. 1). One version (Fig. 1*A*) hereafter termed the “basic” condition consisted of a sequence of brief (25 ms) chords, each containing a random number of pure tone components that varied from 1 chord to the next. Partway through the signal, a certain number of components were fixed across chords for the remaining duration. The second version (Fig. 1*C*) contained loud noise bursts (25 ms) interspersed between successive chords. The noise bursts were intended to break the pattern of repeating tonal components that comprise the figure and reduce possible effects of adaptation, which may underlie figure detection. In previous behavioral experiments (Teki et al. 2013), this manipulation revealed robust figure-detection performance. In fact, listeners continued to detect the “figure” significantly above chance for intervening noise durations of up to 500 ms, demonstrating that the underlying mechanisms, which link successive “temporally coherent” components across time and frequency, are robust to interference over very long time scales.

We used MEG to track, with excellent temporal resolution, the process of figure-ground segregation and the brain areas involved. We observed robust early (within 200 ms of figure onset) evoked responses that were modulated by the number of temporally correlated channels comprising the figure. Sources underlying this bottom-up figure-ground segregation were localized to PT and, the IPS, demonstrating that this area, outside the “classic” auditory cortex, is also involved in auditory scene analysis.

## Materials and Methods

### Participants

Sixteen participants (9 females; mean age: 26.9 years) with normal hearing and no history of audiological or neurological disorders took part in the study. Experimental procedures were approved by the Institute of Neurology Ethics Committee (University College London, UK), and written informed consent was obtained from each participant.

### Stimuli

**Figure 2.**
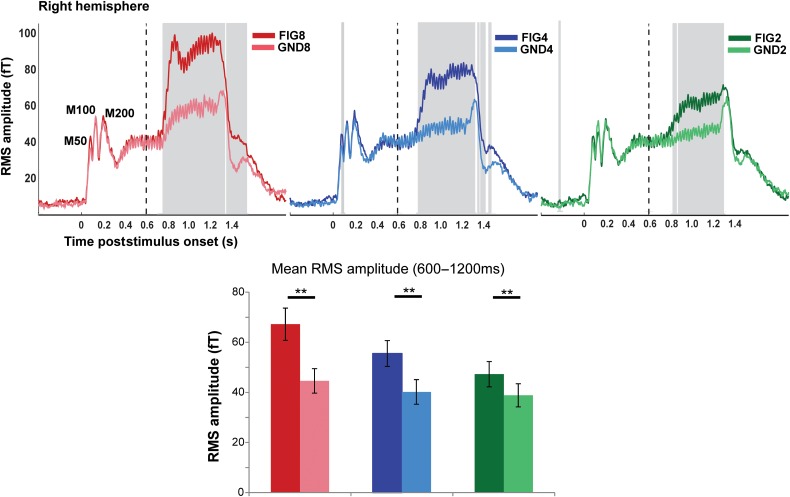
MEG evoked responses to the basic SFG stimulus. (Top) Each plot depicts the group-RMS response to the basic SFG stimulus in the right hemisphere (left hemisphere responses are identical). The onset of the stimulus occurs at *t* = 0 and offset at *t* = 1200 ms, the transition to the figure, as indicated by the dashed vertical lines, occurs at *t* = 600 ms. The responses to the figure and ground segments are shown in the darker and lighter shade of each color: red (FIG8 and GND8), blue (FIG4 and GND4), green (FIG2 and GND2). The shaded gray bars indicate times where a significant difference between the response to the figure and its corresponding control stimulus was observed (based on bootstrap analysis; see Materials and Methods). (Bottom) Mean RMS amplitude in each of the conditions computed over the figure interval (between 600 and 1200 ms poststimulus onset). A repeated-measures ANOVA analysis indicated significant differences between each FIG and GND pair. ** indicates *P* ≤ 0.01.

Signals consisted of a sequence of 25 ms chords, each comprising a random set of tones, drawn from a fixed frequency pool ranging from 0.17 to 2.5 kHz spaced in 1/24 octave steps. This range is narrower than that in our previous studies (0.17–7.2 kHz; Teki et al. 2011, 2013) due to the low-pass filtering characteristics of the Etymotic tubes used for sound delivery. Each chord contained an average of 10 (varying between 5 and 15) pure tone components that changed randomly from 1 chord to the next. A “figure” is incorporated in this randomly varying tonal stimulus by randomly repeating a number of frequencies (“coherence” of the figure: 2, 4, or 8) over a certain number of chords (referred to as the “duration” of the figure). The resulting percept is that of a grouped “auditory object” (“figure”) that pops out from the background. Importantly, the figure is only detectable by integrating the repeating components across frequency and time as the “background” and “figure” components are indistinguishable within a particular chord.

In earlier work, we used a stimulus design where the figure appeared for a brief duration (ranging from 50 to 350 ms) amidst an ongoing random chord sequence (Teki et al. 2011, 2013, 2016). For the present study, the stimulus was modified such that the figure was introduced exactly midway during the stimulus and remained present until offset as shown in Figure 1. This design was used to specifically examine time-locked responses evoked by the appearance of the figure, as well as later activity potentially related to the ongoing representation of the figure amid the fluctuating background.

The stimulus was created by first generating a background-only signal for the total duration of the stimulus and then incorporating additional repeating (“temporally correlated”) tones (2, 4, or 8, hereby referred to as “FIG2”, “FIG4”, and “FIG8”, respectively) during the second half of the signal. Similarly, additional uncorrelated components (2, 4, or 8; randomly varying across chords) were incorporated in the stimuli (50%) that did not contain a figure, to control for the increase in energy associated with the addition of the figure components. These “ground” (or no-figure) signals will be referred to as “GND2”, “GND4”, and “GND8”, respectively. See a schematic representation of FIG4 and GND4 signals in Figure 1. Overall, half of the signals contained a figure (with equal proportions of FIG2, FIG4, and FIG8) and the other half did not (with equal proportions of GND2, GND4, and GND8).

Two versions of the SFG stimuli were used in different blocks: the “basic” version (Fig. 1*A*) consisted of consecutive 25 ms chords (1200 ms long stimulus with the figure appearing at 600 ms post onset); and the “noise” version (Fig. 1*C*) consisted of 25 ms of wide-band white noise interspersed between successive 25 ms long chords (2400 ms long stimulus with the figure appearing at 1200 ms post onset; note that the number of chords is identical to that in the “basic” stimulus). The level of the noise was set to 12 dB above the level of the chords.

All acoustic stimuli were created using MATLAB 7.5 software (The Mathworks Inc.) at a sampling rate of 44.1 kHz and 16-bit resolution. Sounds were delivered binaurally with a tube phone attached to earplugs (E-A-RTONE 3A 10 Ω, Etymotic Research, Inc.) inserted into the ear canal and presented at a comfortable listening level adjusted individually by each participant. The experiment was executed using the Cogent toolbox (http://www.vislab.ucl.ac.uk/cogent.php).

### Procedure

The recording started with a functional source-localizer session where participants were required to attend to a series of 100 ms long pure tones (1000 Hz) for approximately 3 min. A variable number of tones (between 180 and 200) were presented with a random interstimulus interval of 700–1500 ms. Subjects were asked to report the total number of tones presented. This “localizer” session served to identify channels that respond robustly to sound. These were used for subsequent analysis of the sensor-evoked responses to the SFG stimuli.

During the experiment, subjects were engaged in an incidental visual task while passively listening to the SFG stimuli. The visual task consisted of landscape images, presented in a series of 3 (each image was presented for 5 s, with an average gap of 2 s between groups during which the screen was blank). Subjects were instructed to fixate in a cross at the center of the display and press a button whenever the third image in a series was identical to the first or the second image. Such repetitions occurred on 10% of the trials. Responses were executed using a button box held in the right hand. The visual task served as a decoy task—a means to ensure that subjects’ attention was diverted away from the acoustic stimuli. At the end of each block, subjects received feedback about their performance (number of hits, misses, and false positives). To avoid any temporal correlation between the auditory and visual presentation, the visual task was presented from a different computer, independent from the one controlling the presentation of the acoustic stimuli.

The MEG experiment lasted approximately 1.5 h and consisted of 8 blocks. Four blocks involved presentation of the “basic” SFG stimulus, while the “noise” condition was presented in the remaining 4 blocks. The order of the presentation was counterbalanced across subjects. A total of 660 trials were presented for each condition—110 trials for each combination of stimulus type (figure and ground) and number of added components (2, 4, and 8). Each “basic” block took between 8 and 10 min and the “noise” blocks took twice as long. Subjects were allowed a short rest between blocks but were required to stay still.

### MEG Data Acquisition and Preprocessing

Data were acquired using a 274-channel, whole-head MEG scanner with third-order axial gradiometers (CTF systems) at a sampling rate of 600 Hz and analyzed using SPM12 (Litvak et al. 2011; Wellcome Trust Centre for Neuroimaging, London) and Fieldtrip (Oostenveld et al. 2011) in MATLAB 2013 (MathWorks Inc.). The data from the localizer session were divided into 700 ms epochs, including 200 ms prestimulus baseline period, baseline-corrected, and low-pass filtered with a cutoff frequency of 30 Hz. The M100 onset response (Roberts et al. 2000) was identified for each subject as a source/sink pair in the magnetic field contour plots distributed over the temporal region of each hemisphere. For each subject, the 40 most activated channels at the peak of the M100 (20 in each hemisphere) were selected for subsequent sensor-level analysis of the responses evoked by the SFG stimuli.

Data epochs from the main experimental blocks consisted of a 500 ms prestimulus baseline and a 700 ms poststimulus period (overall 2400 ms for “basic” and 3600 ms for “noise” conditions). Epochs with peak amplitudes that deviated from the mean by more than twice the standard deviation (typically ∼7%) were flagged as outliers and discarded automatically from further analyses (∼100 epochs were obtained for each stimulus condition). Denoising Source Separation analysis (DSS, see de Cheveigné and Parra 2014 for an extensive review of the method and its applications) was applied to each stimulus condition to extract stimulus-locked activity (the most reproducible linear combination of sensors across trials)—the 2 most repeatable components in each condition were retained and projected back to sensor space.

Epochs were then averaged and baseline corrected to the prestimulus interval. In each hemisphere, the root-mean-squared (RMS) field strength across 20 channels (selected from the localizer session) was calculated for each participant. The time course of the RMS, reflecting the instantaneous power of neural responses, is employed as a measure of neuronal responses evoked in the auditory cortex. As most of the observed activity (including in the source space) was in auditory cortex, selecting channels based on the M100 represents a reasonable approach for summarizing the sensor-level data in a single time series. For purposes of illustration, group-RMS (RMS of individual subjects’ RMS) is shown, but statistical analysis was always performed across subjects, independently for each hemisphere.

### Statistical Analysis

**Figure 3.**
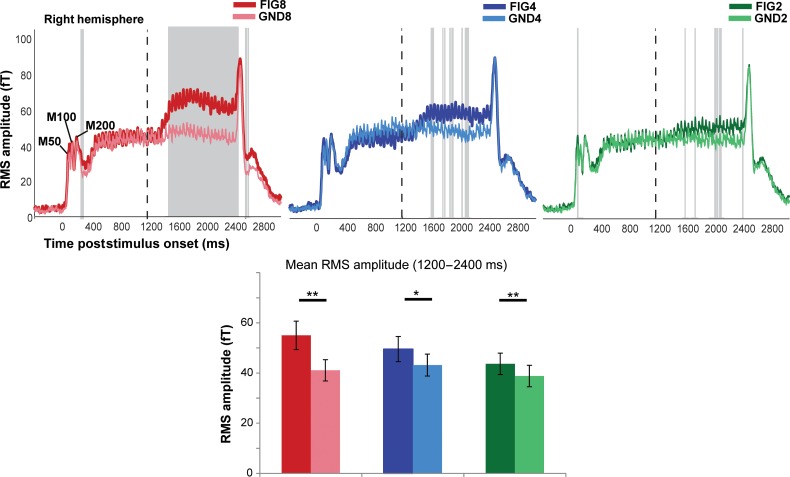
MEG evoked responses to the noise SFG stimulus. (Top) Each plot depicts the group-RMS response to the noise SFG stimulus in the right hemisphere (left hemisphere responses are identical). The onset of the stimulus occurs at *t* = 0 and offset at *t* = 2400 ms, the transition to the figure, as indicated by the dashed vertical lines, occurs at *t* = 1200 ms. The responses to the figure and ground segments are shown in the darker and lighter shade of each color: red (FIG8 and GND8), blue (FIG4 and GND4), green (FIG2 and GND2). The shaded gray bars indicate times where a significant difference between the response to the figure and its corresponding control stimulus was observed (based on bootstrap analysis; see Materials and Methods). (Bottom) Mean RMS amplitude in each of the conditions computed over the figure interval (between 1200 and 2400 ms poststimulus onset). A repeated-measures ANOVA analysis indicated significant differences between each FIG and GND pair. ** indicates *P* ≤ 0.01; * indicates *P* > 0.02.

To estimate the time required to discover the figure, the difference between the RMS waveforms of each FIG and GND pair was calculated for each participant and subjected to bootstrap resampling (2000 iterations; balanced; Efron and Tibshirani 1993). The difference was deemed significant if the proportion of bootstrap iterations that fell above or below zero was >99.9% (i.e., *P* < 0.001) for 5 or more consecutive samples. The first significant sample identified in this way is considered the earliest time point at which the response to the figure differed significantly from the corresponding GND control. The bootstrap analysis was run over the entire epoch duration, and all significant intervals are indicated in Figures 2 and 3 as shaded gray regions.

A repeated-measures ANOVA, with mean amplitude between figure onset and offset (*t* = 600–1200 ms for basic, and *t* = 1200–2400 ms for noise conditions, respectively) as the dependent variable, was used to examine global effects of stimulus (FIG or GND), number of added components (2, 4, and 8), and hemisphere.

### Source Analysis

Source analysis was performed using the generic “Imaging” approach implemented in SPM12 (Litvak et al. 2011; López et al. 2014). We used a classical minimum norm algorithm that seeks to achieve a good data fit while minimizing the overall energy of the sources. In SPM12, this method is referred to as independent identical distribution (IID) as it is based on the assumption that the probability of each source being active is independent and identically distributed (Hämäläinen and Ilmoniemi 1994). The IID method corresponds to a standard L2-minimum norm, which consists of fitting the data at the same time as minimizing the total energy of the sources. Standard processing steps were employed. Specifically, data were first concatenated across blocks for each participant. A generic 8196-vertex cortical mesh template was coregistered (as provided in SPM12 and defined in the MNI stereotaxic space) to the sensor positions using 3 fiducial marker locations (Mattout et al. 2007). We then used a standard single-shell head model for the forward computation of the gain matrix of the lead field model (Nolte 2003). Source estimates on the cortical mesh were obtained via inversion of the forward model with the IID method described above.

The IID model was used to identify distributed sources of brain activity underlying the transition from a background to a coherent figure. The inverse estimates were obtained for all 6 experimental conditions together to allow statistical comparisons between them (“basic” and “noise” blocks had to be analyzed separately due to the different epoch lengths). The inverse reconstruction was performed over the largest possible time window to let the algorithm model all the brain sources generating the response (Litvak et al. 2011). For the “basic” stimuli, the inversion used the entire stimulus epoch (from −300 ms to + 1700 ms, relative to stimulus onset). For the “noise” stimuli, this approach did not work well, because the signal-to-noise ratio of the data is intrinsically much smaller. To confirm this, we compared model evidences in the “noise” conditions with inversion over the entire epoch, and inversion over the stimulus epoch from +1200 ms to +2700 ms. The latter yielded a more accurate inversion for all subjects (the difference in log-model evidences, i.e., log Bayes factor, was >10 for all subjects; Penny et al. 2004) and was therefore used for source localization.

Source activity for each condition was then summarized as separate NIfTI images, by applying the projectors estimated in the inversion stage to the averaged trials (to localize evoked activity), over 2 distinct time windows: an initial transition phase (“early”; a 100 ms period starting from the first time point at which the figure and the corresponding ground became significantly different), as well as a later phase (“late”; a 100 ms period before stimulus offset). The specific values for the time windows used for source localization for each coherence value are detailed in the Results section. The resulting 3D images were smoothed using a Gaussian kernel with 5-mm full-width at half maximum and taken to second-level analysis for statistical inference.

**Figure 4.**
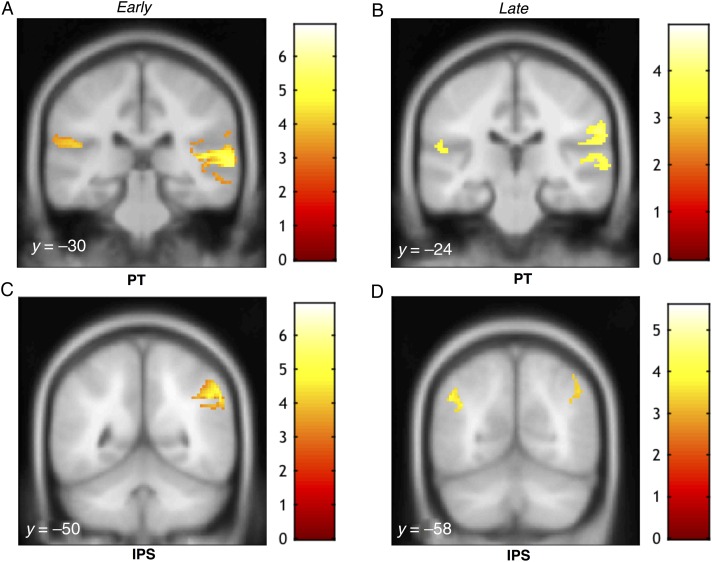
MEG source activations as a function of coherence for the basic SFG stimulus. Activations (thresholded at *P* < 0.001, uncorrected) are shown on the superior temporal plane of the MNI152 template image and the corresponding *y* coordinates are overlaid on each image. The heat map adjacent to each figure depicts the *T* value. Coordinates of local maxima are provided in Table 1. Maximum response during the early transition period was observed in PT and right inferior parietal cortex (*A*) as well as in the right IPS (*C*). Activity during the later response window was observed in PT bilaterally and the right inferior parietal cortex (*B*) as well as in both the left and right IPS (*D*).

At the second level, the data were modeled with 6 conditions (GND8, GND4, GND2, FIG2, FIG4, and FIG8) with a design matrix including a subject-specific regressor and correcting for heteroscedasticity across conditions. We sought to identify brain areas whose activity increased parametrically with corresponding changes in coherence (i.e., over and above the changes in power associated with adding components). For this purpose, a parametric contrast [−8 −4 −2 +2 +4+8]/14 was used. Effectively, the contrast can be expressed as: 2 × (FIG2-GND2) + 4 × (FIG4-GND4) + 8 × (FIG8-GND8), thus targeting brain regions whose activity is parametrically modulated by rising temporal coherence (2 < 4 < 8) while controlling (by subtracting activity of matched GND signals) for the increase in power associated with the added figure components. We also used a simple “Figure versus Ground” contrast: [−1 −1 −1 1 1 1]/3. Statistical maps were initially thresholded at a level of *P* < 0.001 uncorrected, and peaks were considered significant only if they survived family-wise error (FWE) correction at *P* < 0.05 across the whole-brain volume. Because we had prior hypotheses regarding PT and IPS based on our fMRI results (Teki et al. 2011), FWE correction was also applied using small volume correction (SVC; Frackowiak et al. 2003) within these regions. Due to limitations inherent to the resolution of our source analysis, a (conservative) compound mask over PT and the adjacent Heschl's Gyrus (HG) was used. The corresponding cortical masks were determined by reference to the Juelich histologic atlas (for IPS; Eickhoff et al. 2005; Choi et al. 2006; Scheperjans et al. 2008), and the Harvard–Oxford Structural atlases (for Heschl's gyrus and PT; Desikan et al. 2006), available in FSLview (http://surfer.nmr.mgh.harvard.edu/), thresholded at 10% probability. SVC-corrected regions are indicated by asterisks in Tables 1 and 2.

**Table 1:** MEG sources whose activity increased with coherence for the basic SFG stimulus

Brain area	Hemisphere	Response phase	x	y	z {mm}	*t* value
PT	R	Early	52	−18	12	6.23
			64	−18	6	6.12
HG*	R	Early	42	−26	8	4.31
IPS	R	Early	50	−56	34	5.98
			48	−62	28	4.53
IPS	L	Early	−36	−72	42	4.81
			−30	−66	46	4.59
PT*	L	Early	−60	−32	12	3.56
			−58	−30	18	3.46
IPS	R	Late	48	−56	32	5.08
			44	−62	28	3.93
PT*	R	Late	56	−6	12	4.16
			64	−20	8	3.75
PT*	L	Late	−50	−20	14	3.75
			−56	−20	4	3.44

Note: Local maxima are shown at *P* < 0.05 (FWE) at the whole-brain level.

*Small volume-corrected *P* < 0.05 (FWE).

**Table 2:** MEG sources whose activity increased with coherence for the noise SFG stimulus

Area	Hemisphere	Response phase	x	y	z	*t* value
PT*	R	Early	60	−24	20	4.21
			62	−28	6	4.03
PT*	L	Early	−60	−32	24	4.12
			−62	−38	14	3.86
PT	L	Late	−62	−30	24	6.76
			−60	−38	14	5.44
Postcentral gyrus	R	Late	40	−16	38	5.40
			50	−14	42	4.99
PT*	R	Late	54	−26	18	4.34
			64	−34	12	4.15
IPS*	R	Late	30	−40	62	4.18
			28	−46	54	4.13

Note: Local maxima are shown at *P* < 0.05 (FWE) at the whole-brain level.

*Small volume-corrected *P* < 0.05 (FWE).

## Results

The performance on the incidental visual task was at ceiling for all participants, suggesting that they remained engaged in the task throughout the experiment. Since participants were naive to the nature of the acoustic stimuli, and thus unlikely to actively attend to the figures, it can be assumed that the observed auditory responses primarily reflect bottom-up, stimulus-driven processes.

### Basic SFG: Evoked Responses

Figure 2 shows the group-RMS of stimulus-evoked fields, separately for the corresponding FIG and GND conditions, in the right hemisphere (a similar pattern is observed in the left hemisphere). In all conditions, a standard sound onset response is observed with a clear M50, M100, and M200 peak complex (indicated in Fig. 2). The ongoing slow evoked response is characterized by a constant 40 Hz fluctuation of mean evoked power, which follows the rate of presentation of individual chords (every 25 ms).

Following the transition to the figure, clear evoked responses are observed in all FIG conditions. This response consists of an early transient phase characterized by a sharp increase over a few chords (more evident for FIG8 and FIG4), leading to a local maximum in evoked activity, and followed by a more sustained phase until stimulus offset.

The responses to the control GND stimuli allow us to distinguish whether the figure-evoked responses are mediated simply by an increase in energy associated with the additional components or relate specifically to the computation of temporal coherence, linked to the appearance of the figure. Indeed, a transition response (i.e., increase in RMS amplitude as a function of the number of added components) is also present in the GND conditions. However, this response is significantly lower in amplitude and lacks the initial transient phase (sharp increase in power), demonstrating that the response observed for the FIG conditions is largely driven by the temporal coherence of the components comprising the figure.

Bootstrap analysis (see Materials and Methods) revealed that the difference between the response to the figure and its corresponding control condition remains significant throughout the figure segment (indicated by the gray-shaded region), until after sound offset. The first significantly different sample (i.e., the time when the response to the FIG condition first diverges from that to GND) occurred at 158 ms (158 ms), 206 ms (195 ms), and 280 ms (225 ms) posttransition in the left (right) hemispheres for FIG8, FIG4, and FIG2, respectively (see Fig. 2).

A repeated-measures ANOVA with mean amplitude during the figure interval as the dependent variable, and condition (FIG vs. GND), number of components (8, 4, and 2), and hemisphere (left vs. right) as factors indicated no main effect of hemisphere (*F*_1,15_ = 3.25 *P* = 0.091) but confirmed significant main effects of condition: *F*_1,15_ = 58.53, *P* < 0.001, number of added components: *F*_2,30_ =27.26, *P* < 0.001, as well as a significant interaction between condition and number of added components: *F*_2,30_ = 13.25, *P* < 0.001. The interaction indicates that the amplitude of mean evoked field strength is higher for the figure, over and above the effect of increase in spectral energy, and it increases significantly with the number of coherent components in the figure. We refer to this effect as the effect of coherence. A series of repeated-measures *t* tests for each FIG and its corresponding GND (data averaged over hemispheres) confirmed significant differences for all pairs (FIG8 vs. GND8: *t* = 7.01 *P* < 0.001; FIG4 vs. GND4: *t* = 6.77 *P* < 0.001; FIG2 vs. GND2: *t* = 4.25 *P* = 0.01), demonstrating that the brains of naive listeners are sensitive to the temporal coherence associated with only 2 repeating components.

### Noise SFG: Evoked Responses

Figure 3 shows group-RMS of stimulus-evoked fields for the noise SFG stimuli. The general time course of evoked activity is similar to that observed for the basic SFG stimulus. The ongoing slow evoked response is characterized by a constant 20 Hz fluctuation of mean evoked power, which follows the rate of the (loud) noise bursts interspersed between chords.

The addition of a figure is associated with a sharp increase in power, followed by a sustained-like phase that persists until stimulus offset. A bootstrap analysis revealed significantly greater responses to each figure condition compared with its corresponding control, as shown in Figure 3. The latencies at which FIG responses became significantly different from the responses to the GND were approximately 238 ms (300 ms), 720 ms (410 ms), and 412 ms (412 ms) in the left (right) hemisphere for coherence of 8, 4, and 2, respectively.

A repeated-measures ANOVA with mean amplitude during the figure interval as the dependent variable, and condition (FIG vs. GND), number of components (8, 4, and 2), and hemisphere (left vs. right) as factors indicated no main effect of hemisphere (*F*_1,15_ = 3.21 *P* = 0.093) but confirmed significant main effects of condition: *F*_1,15_ = 31.98, *P* < 0.001, number of added components: *F*_2,30_ = 7.28, *P* = 0.003, as well as a significant interaction between condition and number of added components: *F*_2,30_ = 4.55, *P* = 0.019 (effect of figure coherence). A series of *t* tests for each FIG and GND pair (data averaged over hemispheres) confirmed significant differences for all [FIG8 vs. GND8: *t* = 5.02 *P* < 0.001; FIG4 vs. GND4: *t* = 2.4 *P* = 0.024; FIG2 vs. GND2: *t* = 2.84 *P* = 0.012], demonstrating that despite the loud noise interspersed between successive chords (resulting in large power fluctuations across the entire spectrum and therefore reduced power differences between channels) even a figure consisting of only 2 coherent components is reliably encoded by the brains of naive listeners.

A repeated-measures ANOVA (over mean amplitude during the figure period) with block (“basic” vs. “noise”), condition (FIG vs. GND), number of components (8, 4, and 2), and hemisphere (left vs. right) as factors indicated no main effect of block (*F*_1,15_ = 2.5 *P* = 0.128) or hemisphere (*F*_1,15_ = 3.5, *P* = 0.08) but confirmed significant main effects of condition: *F*_1,15_ = 61.3, *P* < 0.001, number of added components: *F*_2,30_= 23.33, *P* < 0.001, as well as an interaction between condition and number of added components: *F*_2,30_ = 15.06, *P* < 0.001 (effect of figure coherence; as observed separately for “basic” and “noise” stimuli). The following interactions were also significant: 1) between block and number of added components *F*_1,15_ = 16.2, *P* = 0.001, 2) between block and condition *F*_2,30_ = 5.23 *P* = 0.01, both due to the fact that the effects of condition and number of components were weaker in the “Noise” relative to the “Basic” stimuli. Crucially however, both stimulus types show similar coherence effects.

### Basic SFG: Source Analysis

To identify brain regions whose activity is parametrically modulated by the coherence of the figure (on top of the increase in power associated with the added figure components), we tested for a signal increase with a parametric contrast over GND8, GND4, GND2, FIG2, FIG4, and FIG8 conditions (see “Materials and Methods”). This contrast mirrors the interaction observed in the analysis of the time domain data and is in line with our previous fMRI study, where significant parametric BOLD responses were observed in the right PT and IPS (Teki et al. 2011). Although the spatial resolution of MEG does not match the high resolution provided by fMRI, recent advances in MEG source inversion techniques permit source localization with a relatively high degree of precision (López et al. 2014).

To capture effects associated with the initial discovery of the figures as well as later processes related to tracking the figures within the random background, we analyzed sources of evoked field strength in two 100 ms time windows: 1) “Early”: starting from the first time sample that showed significant difference between the figure and ground conditions as determined by the bootstrap analysis above (FIG 8: *t* = 158–258 ms ; FIG4: *t* = 195–295 ms; FIG2: *t* = 225–325 ms) and 2) “Late”: during the sustained portion of the response, immediately preceding the offset of the stimulus (i.e., from *t* = 1100–1200 ms).

The results for the early phase revealed robust activations in the PT bilaterally (*P* < 0.05 FWE), and the right inferior parietal cortex bordering the supramarginal gyrus that varied parametrically with the coherence of the figure (Fig. 4*A*; Table 1). We also observed activation in the IPS, and the corresponding activation clusters were clearly spatially separated from the temporal sources, even in the uncorrected *P* < 0.001 *t*-maps (see Fig. 4). We also observed some activity in lateral HG that was contiguous with the PT activity in the right hemisphere only. A separate mask, centered on bilateral medial HG, suggested that coherence-related activity is also observed in the primary auditory cortex. However, due to this being a post hoc analysis, and also because of limits inherent to the resolution of MEG source analysis used here, it is difficult to distinguish this cluster from PT.

Activations in the late phase also involved PT bilaterally and right inferior parietal cortex (*P* < 0.05 FWE; small volume-corrected). We also examined activity in the IPS during both time windows. Figure 4*C*,*D* show significant activation clusters in the IPS (*P* < 0.05 FWE; small volume-corrected) observed during the early and later response phase, respectively. There was no interaction between response phase (“early” or “late”) and coherence, suggesting that IPS and PT contributed to the early and late phase processing in equal measure.

**Figure 5.**
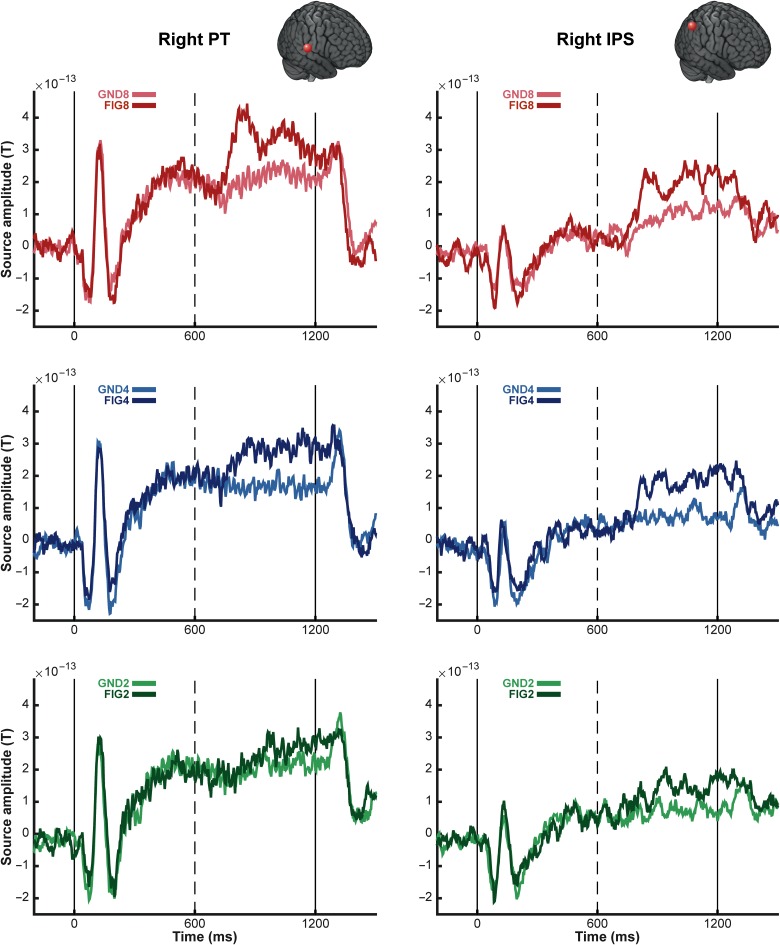
Group average of source activity waveforms for the basic SFG stimuli. The average source activity waveforms for the basic SFG stimuli were computed for sources in the right posterior superior temporal gyrus (MNI coordinates [64, −14, 8]; left panels) and the right intraparietal sulcus (MNI coordinates [54, −50, 40]; right panels). The 6 experimental conditions (FIG and GND; 2, 4 and 8 added components) were inverted together over the entire stimulus epoch, and the corresponding source activity was extracted using the maximum a posteriori (MAP) projector in SPM12 (López et al. 2014). The resulting time-course data were first averaged over trials, then over all available subjects (*N* = 16). The onset (*t* = 0 ms) and offset (*t* = 1200 ms) of the stimulus are marked by solid vertical lines; dashed vertical lines indicate the transition to the figure (*t* = 600 ms). The brain insets in each panel indicate the location of the sources for each region.

Figure 5 shows the group-averaged source waveforms extracted from the right PT and IPS for the basic condition. Both show activation consistent with the sensor-level RMS data (see Fig. 2). The IPS source exhibits weaker onset and offset responses, and lower amplitude sustained activity, consistent with its location further upstream within the processing hierarchy. Importantly, however, the response associated with the appearance of the figure is similar in magnitude in both areas. A repeated-measures ANOVA, with area (PT and IPS) and number of components as factors, was run on the mean amplitude difference between FIG and GND pairs during the figure period (600–1200 ms post onset). This showed a significant main effect of number of components (*F*_2,30_ = 3.70, *P* < 0.05) only. The effect of area was not significant (*F*_1,15_= 0.16, *P* > 0.1), and there was no interaction between factors (*F*_2,30_ = 0.87, *P* > 0.1), confirming that the effect of coherence was equally present in both PT and IPS.

We also conducted simple contrasts of “Figure” versus “Ground” (over the “early” time windows as described above). The negative contrast (“Ground” > “Figure”) was used to address an alternative hypothesis for the mechanisms underlying figure-ground segregation, that is, adaptation-based mechanisms (e.g., stimulus-specific adaptation, SSA; Nelken 2014; Pérez-González and Malmierca 2014), which may be sensitive to repetition within the “coherent” channels. This effect would be observable as a decrease in activation for FIG relative to GND stimuli. However, the relevant contrast yielded no significant activations, both over the entire brain volume and within HG-centered masks. The positive contrast (“Figure” > “Ground”) yielded activations essentially identical to those reported in Figure 4 and Table 1.

### Noise SFG: Source Analysis

We examined the sources of evoked field strength underlying figure-ground processing in the noise SFG stimulus using a 200 ms long window starting from the first time sample that showed significant difference between the figure and its ground control as determined by the bootstrap analysis (FIG 8: *t* = 238–438 ms; FIG4: *t* = 410–610 ms; FIG2: *t* = 412–612 ms), and another 200 ms window, during the later phase of the response, immediately preceding the offset of the figure segment (*t* = 2200–2400 ms). The longer window for source localization in the noise condition (100 ms SFG chords and 100 ms noise) is effectively equal to the 100 ms window (all SFG chords) used for the localization of activations in the basic condition.

**Figure 6.**
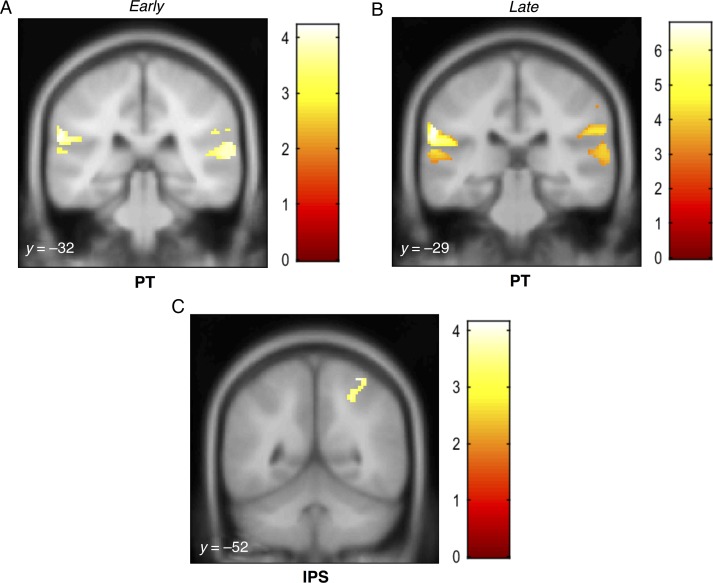
MEG source activations as a function of coherence for the noise SFG stimulus. Activations (thresholded at *P* < 0.001, uncorrected) are shown on the superior temporal plane of the MNI152 template image, and the corresponding *y* coordinates are overlaid on each image. The heat map adjacent to each figure depicts the *t* value. Coordinates of local maxima are provided in Table 2. Maximum response during the early transition period was observed in the right PT and left MTG (*A*). Significant activity during the late response period was observed in the PT bilaterally as well as the right precentral gyrus and rolandic operculum (*B*) and in the left IPS (*C*).

The results for the early phase revealed robust activations in the PT bilaterally (*P* < 0.05 FWE) and the right inferior parietal cortex, including the supramarginal gyrus that varied parametrically with the coherence of the figure (Fig. 6*A*; Table 2). Activations in the late phase (Fig. 6*B*; Table 2) involved PT bilaterally and right inferior parietal cortex (*P* < 0.05 FWE; small volume-corrected). Figure 6*C* shows significant activity in the right IPS (*P* < 0.05 FWE; small volume-corrected), observed during the later response phase only. However, no significant interaction between phase (“early” or “late”) and coherence was found. Thus, despite the fact that the “Noise” condition localization was substantially noisier than that for the basic condition (as also reflected in the weaker sensor-level responses), the results suggest a pattern of activation similar to that for the “basic” condition.

## Discussion

We used MEG to assess the temporal dynamics of stimulus-driven figure-ground segregation in naive, passively listening participants. We used the “Stochastic Figure-ground” (SFG) stimulus—a complex broadband signal, which comprises a “figure”, defined by temporal correlation between distinct frequency channels. The SFG stimulus differs from other commonly used figure-ground signals (Kidd et al. 1994, 1995; Micheyl, Hanson, et al. 2007; Gutschalk et al. 2008; Elhilali, Xiang, et al. 2009) in that the “figure” and “ground” overlap in spectrotemporal space like most natural sounds do, and segregation can only be achieved by integration across frequency and time (Teki et al. 2013).

## Evoked Transition Responses

Our results revealed robust evoked responses, commencing from about 150–200 ms after figure onset, that reflect the emergence of the “figure” from the randomly varying “ground” in the absence of directed attention. The amplitude and latency of these responses varied systematically with the coherence of the figure. Similar effects of coherence (for a coherence level of 8 and 10) were recently reported in an EEG study based on a variant of the “basic” SFG stimulus which used continuous changes in the level of coherence (O'Sullivan et al. 2015). However, they observed much longer latencies (e.g., 433 ms for a ramped SFG figure with a coherence of 8) than those here, possibly due to differences in the stimuli used.

The early transient responses were followed by a sustained-like phase, continuing until figure offset, the amplitude of which also varied systematically with coherence. This general pattern was observed for the basic (Fig. 2) and, remarkably, the noise SFG stimulus (Fig. 3)—where successive chords are separated by loud noise bursts. These results demonstrate that the underlying brain mechanisms, hypothesized to compute temporal coherence across frequency channels (Shamma et al. 2011), are robust to interference with the continuity of the scene, even when listeners were naive and engaged in an incidental visual task.

We additionally show that these transition responses scale with coherence not only at the sensor level but also at the level of the underlying neural sources. As shown in Figure 5, group-averaged source waveforms from the right PT show a similar morphology to the sensor-level transition responses: an initial transient peak is followed by a more sustained response, and the amplitude of these 2 response components varies parametrically with the coherence. Interestingly, group-averaged source responses from the right IPS also show striking coherence-modulated transition responses.

### Neural Substrates of Figure-Ground Segregation

The discussion below is predominantly focused on the localization results from the “basic” condition. The responses for the “noise” condition were overall consistent with the “basic” responses but, as expected, yielded weaker effects.

The approach for identifying the neural substrates underlying the detection of the SFG figures was based on a parametric contrast, seeking brain areas where activity is parametrically modulated by the coherence of the figure. We also investigated a simple “ground > figure” contrast to address the alternative hypothesis that figure pop-out may be driven by frequency-specific adaptation (Nelken 2014). According to this account, the presence of the figure may be detectable as a (repetition-based) decrease in activity within the “coherent” channels. That the ground versus figure contrast yielded no significant activations, and in particular none in the primary auditory cortex where stimulus-specific adaptation is widely observed, suggests that adaptation may not be the principal mechanism underlying figure-ground segregation in the SFG stimulus. This is also in line with behavioral results that show that listeners can withstand significant amounts of interference, such as loud noise bursts up to 500 ms long, between successive chords (Teki et al. 2013).

Using the parametric contrast, we analyzed sources of evoked field strength in 2 different time windows to potentially capture 2 distinct response components: an early transient response reflecting the detection of the figure and later processes related to following the figure amidst the background. We found significant activations in PT (Figs 4*A*,*B* and 6*A*,*B*) in both the early and later stages. This is in agreement with previous human fMRI studies of segregation (Gutschalk et al. 2007; Wilson et al. 2007; Schadwinkel and Gutschalk 2010a, b) based on simple tone streams with different spectral or spatial cues for segregation. The similar pattern of activations in PT for both stimulus conditions suggests a common stimulus-driven segregation mechanism that is sensitive to the emergence of salient targets in complex acoustic scenes.

Teki et al. (2011) did not observe any significant BOLD activation related to figure-ground segregation in primary auditory cortex in the region of medial Heschl's gyrus. Similarly, Elhilali, Ma, et al. (2009) did not find evidence of temporal coherence-based computations in the primary auditory cortex of awake ferrets passively listening to synchronous streaming signals. This could possibly be due to the low spike latencies (∼20 ms) in primary cortex, whereby longer integration windows as observed in secondary and higher order auditory cortices (Bizley et al. 2005; Hackett 2011; Atiani et al. 2014) might be crucial for analysis of temporal coherence across remote cortical ensembles. The present results tentatively indicate some evidence of coherence-related activation in human primary auditory cortex during the early phase, but we cannot exclude the possibility that the observed cluster reflects “spillage” of activity from PT and the issue should be elaborated on with further work. Although how and where the precise computational operations that underlie temporal coherence analysis (Krishnan et al. 2014) are implemented in the brain is not completely clear, it is likely that such operations occur along a processing hierarchy whereby cells in higher order centers abstract temporal responses from lower level processing stages. The present results demonstrate that PT forms part of this network.

We found significant activity in the IPS during both early and late response phases (Figs 4*C,D* and 6*C*). These results are in line with our previous fMRI work where we observed that activity in the IPS increases parametrically with the coherence of the figures (Teki et al. 2011). The finding that IPS activity is modulated systematically by coherence is consistent with earlier work implicating the IPS in perceptual organization of streaming signals (Cusack 2005). Since this area lies outside of the “classic” definition of the “auditory system”, it has previously been suggested that IPS activation may not reflect auditory processing per se but rather relate to attentional effects such as the application of top-down attention (Cusack 2005) or the perceptual consequences of a bottom-up “pop-out process” (Shamma and Micheyl 2010; Teki et al. 2011). Due to the inherently low temporal resolution of fMRI, and hence the lack of precise information regarding the timing of the observed BOLD activations, this conjecture was unresolvable in previous data. Our subjects were naive and occupied by an incidental task and as such it is unlikely that they were actively trying to hear out the figures from within the background. This, together with the finding that coherence-modulated IPS activity is observed at the earliest stages of the evoked response, strongly supports the hypothesis that IPS is involved in the initial stages of figure-ground segregation.

Because the computation of temporal coherence relies on reliable, phase-locked encoding of rapidly evolving auditory information, it is likely that the temporal coherence maps as such are computed in auditory cortex, perhaps in PT. IPS might be involved in reading out these coherence maps or in the actual process of perceptual segregation (encoding the input as consisting of several sources rather than a single mixture). Specifically, IPS may represent a computational hub that integrates auditory input from the auditory parabelt (Pandya and Kuypers 1969; Divac et al. 1977; Hyvarinen 1982) and forms a relay station between the sensory and prefrontal cortex, which associates sensory signals with behavioral meaning (Petrides and Pandya 1984; Fritz et al. 2010). Similar computational operations have been attributed to the parietal cortex in saliency map models of visual feature search (Gottlieb et al. 1998; Itti and Koch 2001; Walther and Koch 2007; Geng and Mangun 2009). Overall our results suggest that IPS plays an automatic, bottom-up role in auditory figure-ground processing, and call for a re-examination of the prevailing assumptions regarding the neural computations and circuits that mediate auditory scene analysis.

## Funding

This work is supported by the Wellcome Trust (WT091681MA and 093292/Z/10/Z). S.T. is supported by the Wellcome Trust (106084/Z/14/Z). Funding to pay the Open Access publication charges for this article was provided by Wellcome Trust.
